# Effect of chikungunya, Mayaro and Una virus coinfection on vector competence of *Aedes aegypti* mosquitoes

**DOI:** 10.1016/j.onehlt.2025.100991

**Published:** 2025-02-07

**Authors:** Tessa M. Visser, Haidong D. Wang, Sandra R. Abbo, Chantal B.F. Vogels, Constantianus J.M. Koenraadt, Gorben P. Pijlman

**Affiliations:** a Laboratory of Entomology, Wageningen University and Research, Wageningen, the Netherlands; b Laboratory of Virology, Wageningen University and Research, Wageningen, the Netherlands; c Department of Epidemiology of Microbial Diseases, Yale School of Public Health, New Haven, USA

**Keywords:** Chikungunya virus, Mayaro virus, Una virus, Coinfection, Vector competence, *Aedes aegypti*, Alphavirus

## Abstract

The mosquito-borne alphaviruses chikungunya (CHIKV), Mayaro (MAYV) and the lesser known Una (UNAV) are currently co-circulating in Latin America, sharing their geographical and ecological niche with the *Aedes aegypti* mosquito. The sylvatic MAYV cycle and the unknown cycle of UNAV could possibly spill over and become urban transmission cycles involving *Ae. aegypti*. Despite their potential impact on public health, we know little about arboviral coinfections in humans, animals or mosquitoes. Especially the effect of coinfections on transmission by *Ae. aegypti* mosquitoes is understudied. We investigated the vector competence of *Ae. aegypti* for single, dual, and triple exposures with UNAV, MAYV and CHIKV, provided simultaneously in an infectious blood meal. Mosquitoes were incubated for ten days at 28 °C and 70 % humidity. After RNA extractions from mosquito bodies and saliva, the presence and relative quantity of each virus in coinfected mosquitoes was determined. We show that *Ae. aegypti* can become infected with all three viruses simultaneously, and transmit at least two alphaviruses in a single mosquito bite after dual or triple infection. Additionally, we show for the first time that *Ae. aegypti* is a competent vector for UNAV, and that dual infections do not influence vector competence. In triple coinfections, however, the total viral load carried by mosquitoes decreases, lowering the transmission potential. Understanding how coinfections affect arbovirus biology and transmission of is essential for assessing public health risks. However, emerging *Ae. aegypti*-vectored arboviruses and coinfections are a One Health concern, as ecological and environmental changes will increasingly drive the geographic distributions of viruses, vectors, and hosts in the future.

## Highlights

• This study explored the effect of alphavirus coinfections on *Aedes aegypti* transmission
• *Aedes aegypti* is a competent vector for Una virus
• *Aedes aegypti* can carry three alphaviruses simultaneously
• At least two alphaviruses can be transmitted in a single mosquito bite
• Coinfection with two alphaviruses does not influence vector competence, but triple coinfections reduce the total viral load in mosquitoes

## 1 Introduction

Mosquito-borne alphaviruses (family: *Togaviridae*) of the Semliki Forest virus complex have caused multiple human disease outbreaks in the Americas in recent years [1]. The virus complex includes, among others, the chikungunya (CHIKV), Mayaro (MAYV) and the newly emerging Una (UNAV) virus. Human infections with CHIKV and MAYV have similar symptoms, as both are characterised by high fever, a rash, and debilitating joint pain that can last for weeks or even years [2]. UNAV is less well-characterised, and even though it has been associated with dizziness, the extent to which it makes people ill is poorly understood [3,4]. It has been found to circulate among humans, for instance, a 2017 study in eastern Panama showed 16.0 % UNAV and 1.2 % MAYV neutralizing antibody seroprevalence across several villages [3]. In addition to these symptoms caused by one virus, some humans are shown to have been coinfected with multiple mosquito-borne viruses [5,6], including a case of an alphavirus coinfection with CHIKV and MAYV [2]. In the case of infections with closely related alphaviruses, accurate diagnosis of patients is complicated. Most detection is done by serology, which cannot distinguish between closely related viruses, let alone coinfections [7]. As a result, UNAV, MAYV, and coinfections are underreported in the human population as most samples are diagnosed as CHIKV only. For example, during the 2014 CHIKV epidemic in Trinidad and Tobago, 278 out of 666 acute samples sent for CHIKV investigation were indeed CHIKV positive, while 258 were dengue virus (DENV) positive, and nine were MAYV positive [8]. This study, along with others, show that many regions in South America experience not just outbreaks of a single arbovirus, but multiple co-circulating arboviruses [[8], [9], [10]]. Despite the potential challenge to public health, little is known about the effect of alphavirus coinfection on transmission dynamics and thus disease progression in human and mosquito populations [11].

Depending on the virus species, alphaviruses are transmitted by different mosquito vector species and maintained in different transmission cycles. CHIKV is maintained in an urban cycle, relying on *Aedes aegypti* as key vector [12]. This species occurs globally in temperate regions across the tropical hemisphere and is considered an efficient virus vector between humans due to its strong anthropophilic biting behaviour and breeding in nearby man-made water containers [13]. In contrast to CHIKV, MAYV is mainly maintained in a sylvatic cycle with mosquitoes of the *Haemagogus* genus, such as *Haemagogus janthinomys* [14]. *Haemagogus* mosquitoes are primarily present in Amazonian rainforests. Reservoir hosts of MAYV include several vertebrate hosts such as non-human primates, rodents, birds, sloths and other small mammals [14]. The transmission cycle of UNAV is unknown, but presumably sylvatic [15].

**Fig. 1:**
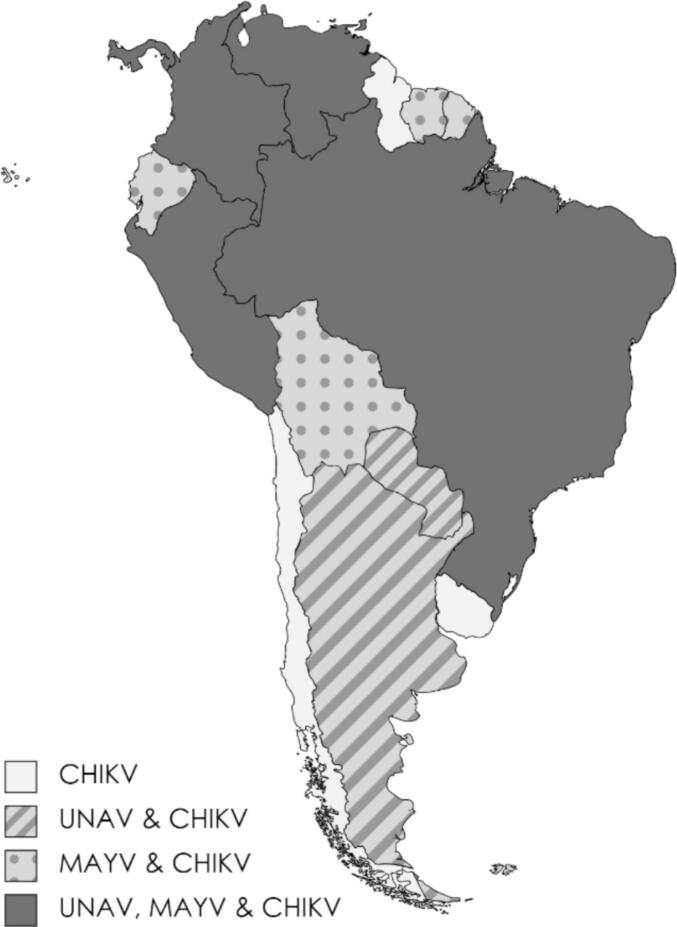
UNA, MAYV and CHIKV distribution on the South American continent. The map depicts countries with past or current human cases or detection in mosquitoes and non-human primates of UNAV, MAYV and CHIKV. It was generated using the free tool https://mapchart.net/ and is based on data provided by PAHO, WHO, and a literature review (for references used, see Table S6).

Regarding *Ae. aegypti*, there are concerns that this urban species may become responsible for the transmission of novel viruses, other than CHIKV [16]. The sylvatic MAYV and the unknown cycle of UNAV could possibly spill over and become urban. Thus far, the three viruses have been detected in humans, primates, and mosquitoes across Latin America (Fig. 1). In fact, the geographical and ecological overlap of the three viruses is increasing. This is partly due to the exploitation of rainforests, which increases contact between humans, animal reservoirs, and mosquito species [17,18]. For instance, hunting in forests was the main risk factor found for MAYV and UNAV infection in the Peruvian Amazon [19]. In addition to introducing infections into urban areas where *Ae. aegypti* is present, such activities may inadvertently facilitate the migration of mosquitoes into new territories. This is for example illustrated by the spread of *Ae. aegypti* populations along the riverways of the Peruvian Amazon using boats and shipping routes [20,21]. Furthermore, MAYV cases have been found in urban areas on the island of Trinidad, where patients did not have a travel history in visiting sylvatic areas, suggesting local transmission [8]. In addition, several laboratory studies have found that *Ae. aegypti* is a competent vector for MAYV [[22], [23], [24]]. These findings emphasise the interconnectedness of animal and human health and the environment, suggesting this is a matter of One Health, rather than just a public health concern. It is therefore critical to investigate these viruses and their vectors to improve preparedness for future disease outbreaks. We hypothesise that *Ae. aegypti* could play a role in UNAV transmission due to the close homology between UNAV, MAYV and CHIKV and the fact that UNAV has been isolated from several mosquito species, including *Aedes* spp. [15].

The detection of coinfections in field-caught mosquitoes is rarely reported. Only one study reported a wild *Aedes albopictus* to be coinfected with CHIKV and dengue virus in Gabon, Central Africa [25]. While this could reflect a lack of coinfections in mosquitoes, it is more likely coinfections are undetected because mosquitoes are often tested in pools rather than individually. Moreover, the detection limit of virus infections of mosquitoes in the field is usually low, sometimes only 1:1000 tested mosquitoes or even less [11,26,27]. Therefore, laboratory studies are needed to examine the possibility of coinfections with alphaviruses and their effect on transmission.

Coinfections in mosquitoes can result in five potential outcomes: enhancement of development of both viruses, inhibition of both viruses, one virus affecting the other virus (and vice-versa), or no effect on either virus [11]. Multiple laboratory studies have examined coinfections in mosquitoes [[28], [29], [30]]. When coinfecting *Ae. aegypti* mosquitoes with a combination of an alphavirus and a flavivirus, there seems to be no or minimal effect on transmission rates [[29], [30], [31]]. This might be due to viruses from different viral families using different replication sites in the cell [30]. Alphavirus coinfections in mosquitoes are expected to have a more profound effect on transmission rates as they use the same endosomal replication sites [32]. This means alphaviruses are more likely to be in direct competition with each other for the same resources. In addition to investigating the vector competence of *Ae. aegypti* for UNAV, we, therefore, examined to what extent alphavirus coinfections influence their transmission by *Ae. aegypti* compared to single infections. We hypothesise that alphavirus coinfections will result in lower transmission rates and overall lower viral load in mosquitoes compared to single infections.

## 2 Materials & methods

### 2.1 Mosquitoes

Female *Ae. aegypti* mosquitoes (Rockefeller strain, obtained from Bayer AG, Monheim, Germany) were used in all experiments. For colony maintenance, adult mosquitoes were kept in 30 cm cubic rearing cages (Bugdorm, Taiwan, China) at 27 ± 1 °C with a 12:12 light: dark cycle and 70 % relative humidity (RH). Mosquitoes were provided with 6 % ad libitum glucose solution and human blood (Sanquin Blood Supply Foundation, Nijmegen, the Netherlands). Blood was provided through Parafilm using a Hemotek PS5 feeder (Discovery Workshops, Lancashire, United Kingdom). Adults laid their eggs on wet filter paper in small plastic cups filled with tap water. One-week-old dried eggs were brushed off the filter paper into trays filled with tap water to which three droplets of Liquifry nr. 1 (Interpet, UK) were added. Hatched larvae were fed daily with TetraMin Baby fish food (Melle, Germany). Newly emerged adults were transferred into rearing cages or set aside in cages for experimental use. Female mosquitoes were kept together with males for three to six days in the experimental cages before females were transferred to experimental buckets (diameter: 12.2 cm, height: 12.2 cm; Jokey, Wipperfürth, Germany) and transported to the Biological Safety Level 3 laboratory for virus infection assays.

### 2.2 Cell culture

African green monkey kidney Vero E6 cells were cultured as monolayers in T25 cell culture flasks (Greiner Bio-One, Kremsmünster, Austria) in HEPES-buffered Dulbecco's Modified Eagle Medium (DMEM, Gibco, Carlsbad, CA, USA) supplemented with 10 % fetal bovine serum (FBS, Gibco), penicillin (100 U/ml, Sigma-Aldrich, Saint Louis, MO, USA) and streptomycin (100 μg/ml, Sigma-Aldrich) (P/S), hereafter called DMEM-supplemented, at 37 °C with 5 % CO_2_. *Aedes albopictus* C6/36 cells were also cultured as monolayers in T25 flasks, in Leibovitz L-15 medium (Gibco) supplemented with 10 % FBS, 2 % tryptose phosphate broth (Gibco) and 1 % nonessential amino acids (Gibco) at 28 °C, hereafter called Leibovitz-supplemented.

### 2.3 Viral stocks

An infectious clone derived from CHIKV strain 37997 (GenBank accession number, EU224270.1) was prepared as described [14]. The first passage was stored in aliquots at -80 °C until further use. The MAYV BeH407 (GenBank accession number, MK573238.1) and UNAV CoAr2380 strains were obtained from the World Reference Center for Emerging Viruses and Arboviruses (University of Texas Medical Branch, Galveston, TX). Passage 2 stocks of all viruses were propagated on C6/36 cells and used in all experiments. Virus titers of stocks were determined by endpoint dilution assays (EPDAs) on Vero cells. All procedures involving infectious viruses were executed in the Biological Safety Level 3 laboratory of Wageningen University & Research.

### 2.4 Infectious blood meal

To stimulate blood feeding of *Ae.* a*egypti* females, the glucose solution was replaced by water a day before the experiments. Infectious blood meals were prepared by diluting the viruses in Leibovitz-supplemented (Gibco) to 200 μl, after which 1300 μl human blood was added to obtain a concentration of 2 × 10^7^ TCID_50_/ml. One ml of the infectious blood meal was offered through Parafilm using the Hemotek PS5 feeder, and the other 500 μl was stored in at -80 °C to determine viral titres of the initial blood meal. The virus concentrations were kept the same for all different treatments: single, dual and triple infections, meaning that a dual-infected mosquito was exposed to 4 × 10^7^ TCID_50_/ml and a triple-infected mosquito with 6 × 10^7^ TCID_50_/ml. Mosquitoes were allowed to feed for one hour ad libitum in light conditions, at 26 °C and 60 % RH. After blood feeding, mosquitoes were anaesthetised with 100 % CO_2_, placed on a porous CO_2_ pad and fully engorged females were selected. Three mosquitoes per single-infection replicate were immediately frozen at -80 °C after selection to determine the amount of virus ingested by the mosquitoes. Exposed mosquitoes were maintained at 28 °C with 6 % glucose solution ad libitum. The glucose solution was refreshed every 2–3 days until ten days post-infection (dpi).

### 2.5 Salivation assay

At ten dpi, mosquitoes were anaesthetised with 100 % CO_2_ and placed on a CO_2_ pad. Mosquitoes that died within the 10-day incubation period were discarded. The remaining females were immobilised by removing their legs and wings with forceps. Then, the proboscis of each mosquito was inserted into a 200 μl yellow pipet tip (Greiner Bio-One) containing five μl of a 1:1 solution of 50 % glucose solution and FBS for 45 min. After salivation, the mosquito bodies were added to 1.5 ml Safe-Seal micro tubes (Sarstedt, Nümbrecht, Germany) containing 0.5 mm zirconium beads (Next Advance, Averill Park, NY, United States). Each saliva sample was also added to 1.5 ml microtubes (Sarstedt) containing 55 μl DMEM-supplemented with additional fungizone (50 μg/ml; Invitrogen), and gentamycin (50 μg/ml; Life Technologies), hereafter called DMEM-complete. Mosquito bodies and saliva samples were stored at -80 °C until further processing.

### 2.6 Infectivity assay

Mosquito bodies were homogenised using a previously described protocol [30]. Briefly, frozen bodies were placed in a Bullet Blender storm (Next advance), resuspended in 100 μl DMEM-complete, and blended a second time. The mosquito saliva samples were thawed at room temperature. Of both the saliva and body homogenate 30 μl was used to inoculate Vero cells in a 96 wells plate. After three hours, the inoculum was removed and replaced with 100 μl DMEM-complete. Cytopathic effects (CPE) were scored at 3 and 6 dpi for all samples. CPE-positive mosquito and saliva samples were selected for RNA extractions and SYBR qRT-PCR. Bodies and salivas of 15 randomly chosen single-infected mosquitoes were titrated using EPDAs.

### 2.7 RNA extraction

TRIzol reagent (Invitrogen, Carlsbad, CA, USA) was used for total RNA isolations from cells and mosquito body homogenates. The Mag-Bind Viral RNA 96 kit (Omega Bio-tek, Norcross, GA, USA) was used to isolate RNA from mosquito saliva samples. Both protocols were used according to the manufacturer's instructions. The samples' RNA yields were determined by a DeNovix DS-11 FX spectrophotometer (DeNovix Inc., Wilmington, DE, USA).

### 2.8 qRT-PCR

To detect UNAV, MAYV and CHIKV in mosquito body and saliva samples, primers targeting the E1 envelope gene were newly designed and synthesised (Eurofins Genomics GmbH, Ebersberg, Germany)(Table S1). First, degenerate primers were used to sequence part of the E1 gene of UNAV and MAYV. Next, specific primers were designed based on the received sequences. CHIKV primers were designed using the available GenBank sequence (Table S1). T7 RNA standards of UNAV, MAYV and CHIKV were generated based on a ∼ 200 bp amplicon using the T7 RiboMAX Kit (Promega, Madison, WI, USA). The in vitro transcript viral RNA was quantified by the DeNovix spectrophotometer and used to make a 10-time dilution series. Viral genome copies were then calculated by an online tool (https://nebiocalculator.neb.com/). The SYBR qRT-PCR reaction was performed in a CFX96 Real-Time PCR instrument (Bio-Rad Laboratories, Hercules, CA, USA) with a 20 μl reaction mix using the iTaq Universal SYBR Green One-Step Kit (Bio-Rad Laboratories). The amplification efficiency (AE) of the three primer sets was comparable between the single, dual and triple virus samples (Fig. S1); however, some high Ct cross-reactivity was observed (Table S2). Therefore, positive UNAV, MAYV and CHIKV samples were determined by checking the qRT-PCR-product specific melt-curve temperatures (UNAV: 81–82.5 °C, MAYV: 83 °C, and CHIKV: 83,5–84,5 °C) which proved to be robust and was confirmed by sequencing PCR products of all different viral treatments, and negative controls. If a sample had a corresponding melt-curve temperature and a Ct value one entire cycle lower than the NTC, it was scored positive and included in the analyses. Based on the abovementioned protocol, every qPCR plate had a standard curve and an individual cut-off value. Based on the specificity test (Table S2), the detection limit roughly corresponds to approximately 2600 copies of UNAV RNA, 910 copies of MAYV RNA, and 490 copies of CHIKV RNA.

### 2.9 Data analysis

First, all data were checked for normality by evaluating the Shapiro-Wilk statistic. To compare infection- and transmission rates and genome copy numbers we used Kruskal-Wallis (KW) tests with Dunn's post-hoc and Bonferroni corrections for multiple comparisons. Effects for all tests were considered significant when *p* < 0.05. All analyses were performed using SPSS statistical software (version 29, IBM Corporation, New York, USA). Figures were made using GraphPad Prism 8.

## 3 Results

**Fig. 2:**
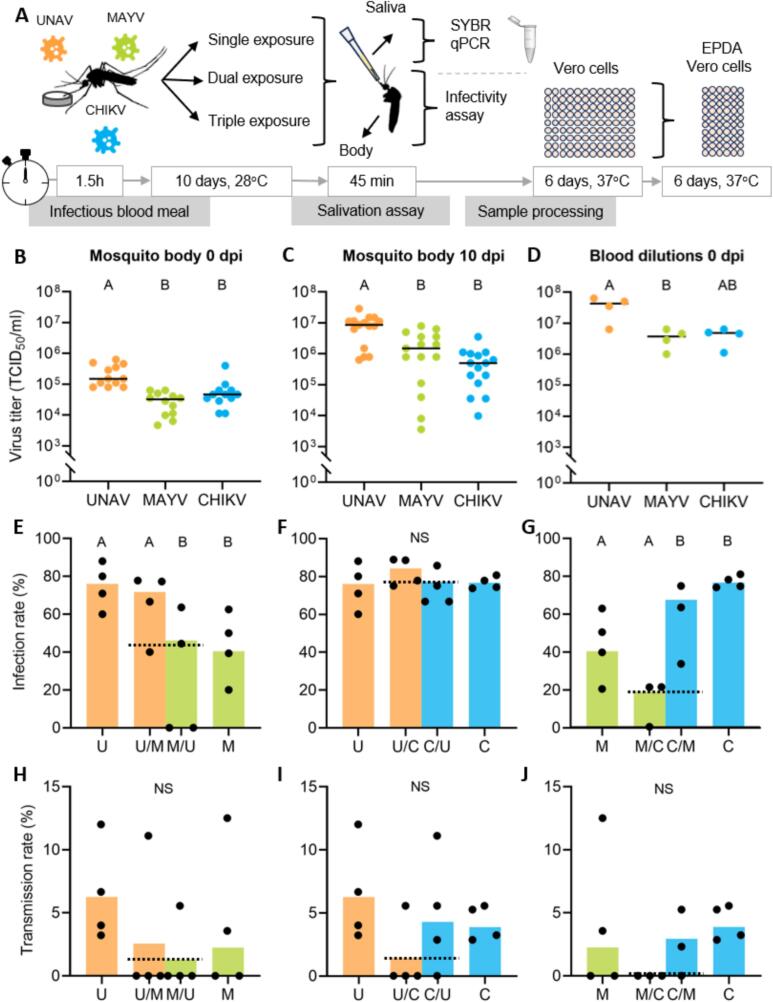
Infection and transmission rates of *Ae. aegypti* mosquitoes infected via blood meal with UNAV, MAYV, CHIKV, or dual combinations of these viruses. (A) Experimental workflow. Female mosquitoes were given an infectious blood meal containing a single, dual, or triple virus combination of UNAV, MAYV, or CHIKV. After ten days of incubation, saliva and body samples were collected and stored at -80 °C until further processing. Next, infection and transmission rates plus viral titers (on a selection of single-infection samples) were determined by infectivity- and End Point Dilution Assays (EPDAs). Then, qRT-PCR on the same samples determined the presence and relative RNA quantity of each virus in coinfected mosquito and saliva samples. Results in all figures were evaluated with a Kruskal-Wallis test and Dunn's post hoc with Bonferroni corrections. Significant differences between treatments (*p* < 0.05) are indicated by different letters. (B) Ingested virus titers of *Ae. aegypti* immediately after blood-feeding from a single-virus source in four independent replicate experiments. Each dot represents one mosquito body. The horizontal bars represent median titers. (C) Virus titers of 15 randomly selected *Ae. aegypti* females at ten dpi. Each dot represents one mosquito body. The horizontal bars represent median titers. (D) Viral titers were determined in the single virus-blood meals of four independent replicates. The horizontal bars represent median titers. (E-G) Infection and (H-J) transmission rates of *Ae. aegypti* mosquitoes at ten dpi determined by qRT-PCR, presented as the percentage of the total number of engorged mosquitoes. Please note that single infection data was used in two panels. This was corrected for (Bonferroni). The single letters, U (UNAV), M (MAYV), and C (CHIKV) correspond with single infected mosquitoes. The two-letter combinations denote mosquitoes exposed to two viruses; the first letter indicates the virus shown on the graph, the second shows the context of the co-exposed virus. The dashed line indicates the percentage of coinfected mosquitoes and saliva samples in dual challenged mosquitoes. Shown are the means of four independent replicates which are depicted with the black dots, except for the MAYV/CHIKV coinfection experiment, which has three replicates. The sample size ranged from 63 to 103 female mosquitoes per treatment.

To investigate the ability of UNAV to infect the bodies and salivary glands of *Ae. aegypti* mosquitoes and to examine the effect of alphavirus coinfections on infection and transmission by *Ae. aegypti,* mosquitoes were given a single, dual, or triple infectious blood meal with all possible combinations of UNAV, MAYV, and CHIKV (Fig. 2A). The experiments were repeated four times unless otherwise stated. After ten days of incubation, mosquito saliva and body samples were collected and stored at -80 °C until further processing. The infection and transmission rates were determined by infectivity assays on Vero cells. Moreover, viral titers were determined by End Point Dilution Assays (EPDAs) on a selection of single-infection samples based on the infectivity assay results. Then, virus-specific qRT-PCR was performed on the same samples to determine the presence and relative quantity of each virus in single infected and coinfected mosquito body and saliva samples.

### 3.1 Aedes aegypti is a competent vector for UNAV transmission

To evaluate the susceptibility of female *Ae. aegypti* mosquitoes for UNAV compared to MAYV and CHIKV, individuals were orally exposed to an infectious blood meal. To check the viral titers after blood-feeding, three fully engorged mosquito bodies were frozen directly after feeding and virus titers were determined by EPDA (Fig. 2B). The ingested viral titers show that MAYV and CHIKV-exposed mosquitoes took up less virus than UNAV-exposed mosquitoes (KW: χ^2^ = 19.208, df = 2, *p* < 0.001 and χ^2^ = 13.792, df = 2, *p* < 0.01 respectively; Fig. 2B). Ten days after the blood meal, a subset of 15 single-infected females per virus treatment (including samples from all experimental replicates) was used to determine the viral body titers by EPDA (Fig. 2C). UNAV-infected mosquitoes had a higher body titer than MAYV and CHIKV (KW: χ^2^ = 13.400, df = 2, *p* < 0.05 and χ^2^ = 20.300, df = 2, p < 0.001 respectively). These observations led us back to titrate the bloodmeal which was also frozen directly after making the virus/blood dilution. The MAYV titers in the blood meal were slightly lower than calculated beforehand and differed from UNAV (Kruskal-Wallis test (KW): χ^2^ = 6.125, df = 2, *p* = 0.046; Fig. 2D). The mosquitoes received a mean UNAV titer of 3.9 × 10^7^ TCID_50_/ml, a mean MAYV titer of 3.7× 10^6^ TCID_50_/ml, and a mean CHIKV titer of 4.3 × 10^6^ TCID_50_/ml (Fig. 2D).

The infection and transmission rates were calculated based on presence of virus in the body or saliva, respectively, using an infectivity assay on Vero cells. The infection rate of UNAV-infected mosquitoes was 76 %, and the transmission rate was 14 % (Table S3). The infection and transmission rates for MAYV were 57 % and 14 %, and for CHIKV, 94 % and 12 % respectively (Table S3). Most importantly, these results show that *Ae. aegypti* is a competent vector for UNAV.

### 3.2 Aedes aegypti can simultaneously transmit at least two alphaviruses

Coinfections of arboviruses can affect the transmission potential of mosquito vectors and may result in the enhancement or inhibition of one or more viruses [11]. To investigate the effect of alphavirus coinfections on the infection and transmission of UNAV, MAYV, and CHIKV, female *Ae. aegypti* mosquitoes were offered a blood meal containing single viruses as described and analysed in the previous paragraph. In addition, dual- and triple-infections were performed concurrently (Fig. 2A). At ten dpi, saliva was collected from the mosquitoes, and infection and transmission rates were determined by infectivity assay on Vero cells (Table S3–5) and by qRT-PCR (Fig. 2 E-J).

**Fig. 3:**
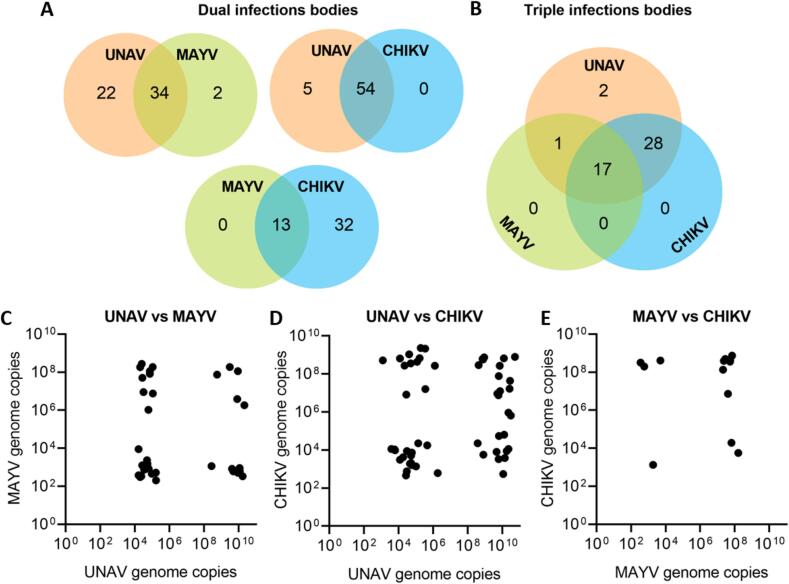
Venn diagrams depicting the number of positive infected mosquito bodies after a coinfectious blood meal detected by qRT-PCR and viral genome copies plotted against each other for dual-infections. All combinations of UNAV, MAYV and CHIKV were provided to *Ae. aegypti* females through a blood meal. The results show the cumulative number of positive bodies for four independent replicates of (A) dual and (B) triple infections. Moreover, we show genome copies per 100 ng RNA material in mosquito bodies from (C) UNAV and MAYV coinfected individuals, (D) UNAV and CHIKV coinfected individuals and (E) MAYV and CHIKV infected individuals.

When mosquitoes were exposed to both UNAV and MAYV, the infection rates were not significantly different compared to infection rates of UNAV and MAYV single-exposure (KW: *p* > 0.05; Fig. 2E). Likewise, compared to single exposure, neither UNAV nor CHIKV infection rates were significantly affected by UNAV and CHIKV co-exposure (KW: p > 0.05; Fig. 2F). MAYV and CHIKV infection rates were also bot not affected by MAYV/CHIKV co-exposure (KW, p > 0.05; Fig. 2G). High proportions of coinfections were found after co-exposure of the different viruses (dashed lines in Fig. 2E-G; numbers in Fig. 3A for dual-exposed and Fig. 3B for triple-exposed mosquitoes).

Coinfection did not significantly affect transmission rates in any of the combinations tested (Fig. 2H-J). Importantly, we confirmed one saliva-positive mosquito for UNAV/MAYV and one saliva-positive mosquito for UNAV/CHIKV by qRT-PCR (Table 1). We know these samples contain infectious viruses, as they were first screened in an infectivity assay, but we were not able to confirm the presence of infectious virus for both. These results show that *Ae. aegypti* can potentially transmit both UNAV and MAYV, and UNAV and CHIKV in a single bite. Unfortunately, several saliva samples that were positive in the infectivity assay on Vero cells, were not confirmed by qRT-PCR (Table 1). In summary, simultaneous exposure can lead to concurrent transmission of alphaviruses without affecting their infection and transmission rates in *Ae. aegypti*.

**Table 1:** qRT-PCR-positive saliva samples versus CPE-positive saliva samples after coinfections. The single letters, U (UNAV), M (MAYV), and C (CHIKV) correspond with single infected mosquitoes. The two-letter combinations denote mosquitoes exposed to two viruses. There were no triple infected saliva samples.

Treatment	qRT-PCR single			qRT-PCR dual			qRT-PCR total	CPE total
*UNAV/MAYV*	*U*	*M*		*U/M*				
	1	0		1			2	14
*UNAV/CHIKV*	*U*	*C*		*U/C*				
	0	2		1			3	9
*MAYV/CHIKV*	*M*	*C*		*M/C*				
	0	2		0			2	9
*UNAV/MAYV/CHIKV*	*U*	*M*	*C*	*U/M*	*U/C*	*M/C*		
	3	0	2	0	0	0	5	6

### 3.3 Viral load in mosquitoes is negatively affected by triple infection

**Fig. 4:**
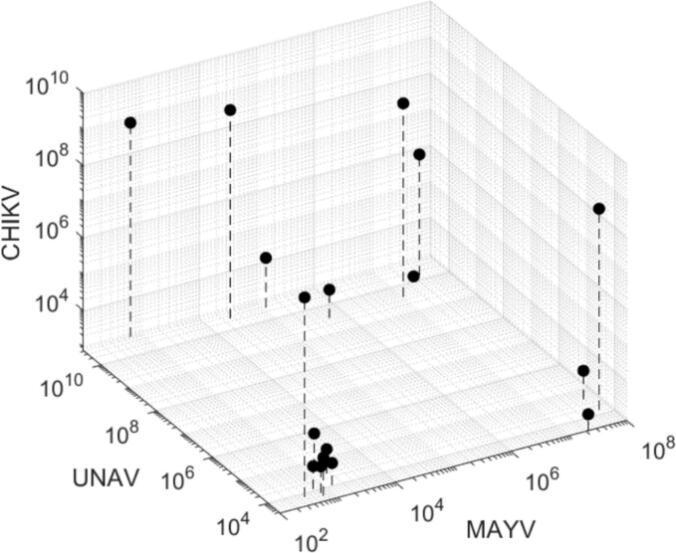
Viral genome copies plotted against each other for triple infections. Shown are genome copies per 100 ng RNA material in mosquito bodies of triple infected mosquitoes. Triple-exposed mosquitoes which resulted in single- or coinfections are excluded from this plot.

**Fig. 5:**
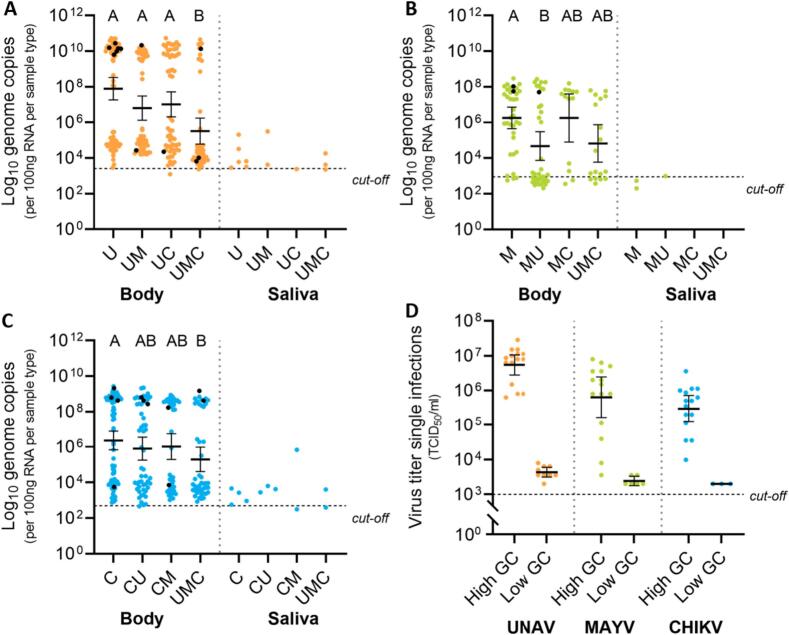
Viral genome copies of UNAV, MAYV and CHIKV-infected mosquitoes after a single, dual or triple virus-infected blood meal. (A) UNAV, (B) MAYV, and (C) CHIKV RNA levels in mosquito bodies and saliva. The single letters, U (UNAV), M (MAYV), and C (CHIKV) correspond with single infected mosquitoes. The two- and three-letter combinations denote mosquitoes exposed to multiple viruses at the same time. Black dots represent the bodies that also resulted in a positive saliva sample. The bars indicate the geometric mean, and the error bars represent the 95 % confidence interval. Results regarding the bodies were evaluated with Kruskal-Wallis tests and a Dunn's post hoc with Bonferroni corrections for multiple comparisons. Significant differences between treatments (*p* < 0.05) are indicated by different letters. (D) Viral titers of 15 mosquito bodies with a ‘high’ genome copy (GC) number and a ‘low’ GC number.

Although coinfection does not seem to significantly influence infection and transmission rates, there may be an effect on the viral load in either the mosquito body or the saliva due to interference or exclusion that could still have a negative effect on virus transmission. Therefore, viral load was determined by qRT-PCR and presented as relative genome copies per 100 ng RNA material at ten dpi for both mosquito bodies and saliva of mosquitoes with a CPE-positive body and CPE-positive saliva sample. A similar pattern is observed across all three dual infection combinations and the triple infections when looking at the genome copies in co-exposed and coinfected mosquitoes plotted against each other (Fig. 3C-D, Fig. 4). Every possible outcome is present in the samples: (1) both viruses are present in low quantity, (2) one virus has a low quantity, the other a high quantity or (3) vice versa, and (4) both viruses are present in a high quantity. Bivariate Pearson correlation tests and linear regressions among the possible virus combinations were all not significant (p > 0.05; Fig. 3C-D). There is a split in RNA quantity in mosquito body samples (Fig. 5A-C), with on one side a group with higher genome copy numbers and on the other a group with lower numbers. Most, but not all, positive saliva samples came from bodies with relatively higher genome copy numbers (black dots depicted in Fig. 5A-C). In general, a decrease in viral load is observed in triple samples compared to single-infected mosquitoes (KW, Dunn's post-hoc: *p* < 0.05; Fig. 5A-C). To investigate if a ‘high’ or ‘low’ genome copy number also corresponds to a higher or lower viral titer, a selection of 15 individuals with high genome copy numbers and 15 individuals with low genome copy numbers were titrated, and this is indeed the case (Fig. 5D). These results show that coinfection with dual virus does not affect the transmission potential of *Ae. aegypti* for either virus. However, the viral load is decreased in mosquitoes with triple infections compared to the other treatments.

## 4 Discussion

There is increasing evidence that humans can become co-infected with various combinations of MAYV, CHIKV, ZIKV, and DENV [2,5,6,9]. While observations of multiple infections in the same patient indicate that *Ae. aegypti* may be exposed to two viruses at the same time, it is unclear if such infections are the result of a single bite from a coinfected mosquito or the result of sequential bites of single-infected mosquitoes. The aim of this study was to determine if *Ae. aegypti* can concurrently transmit UNAV, MAYV, and CHIKV and to explore if coinfections with these alphaviruses affect transmission of each virus. As opposed to our hypothesis, our results show that coinfection with two viruses does not influence the transmission potential of *Ae. aegypti* for either virus. The viral load, however, is decreased in mosquitoes with triple infections compared to the other treatments. In addition, we show that *Ae. aegypti* can transmit two alphaviruses simultaneously, having detected a UNAV- and MAYV-positive saliva sample and a UNAV- and CHIKV-positive saliva sample containing infectious virus.

Other studies investigating flavivirus and alphavirus coinfection in *Ae. aegypti* also report on the possibility of simultaneous transmission of arboviruses [[29], [30], [31]]. This includes one study on MAYV and CHIKV that showed that both viruses could be transmitted equally efficiently as in single infections [33] and another showing the presence of three viruses, DENV, ZIKV and CHIKV in one single saliva sample [29]. Some studies of coinfections, however, show inhibitory effects for one virus. For example, MAYV had a negative impact on dissemination of ZIKV, but not on transmission [34]. Conversely, other studies report enhancing effects such as higher replication of DENV-2 in mosquitoes coinfected with CHIKV [35]. However, two studies investigating simultaneous DENV-2 and CHIKV infections did not find such evidence from coinfected mosquito bodies [36] or saliva samples [37]. In other words, the observed effects seem to be highly dependent on mosquito and viral species used in these studies, and there is thus no clear consensus on general mechanisms behind simultaneous coinfections of arboviruses. This may be the result of the fact that experimental methods of infection and detection of coinfections vary, making it difficult to interpret and compare results. Our study revealed no exclusion, no inhibition, and no enhancement of two alphaviruses in their transmission by *Ae. aegypti* for all three virus combinations. We do see a split in genomic copy numbers in mosquito body samples, which can possibly be explained by mosquitoes with fully disseminated infections versus mosquitoes with local/midgut infections only. Moreover, when exposing mosquitoes to the combination of three alphaviruses, we observed a decrease in viral load for UNAV, MAYV and CHIKV. The reduction in viral load was, for example, also seen in a study with USUV and West-Nile virus coinfected *Culex pipiens* mosquitoes [28]. Eventually, the decrease in viral load in triple infected mosquitoes may negatively impact the amount of virus in the mosquito's saliva, thereby lowering the transmission potential.

In contrast with our work on whole individual mosquitoes, alphaviruses can show superinfection exclusion (SIE) in mosquito cell lines [38]. SIE is an interesting phenomenon whereby a cell that is infected by one virus becomes resistant to infection by another virus. The fact that we did not observe viral interference in the dual-infected mosquito bodies could be due to UNAV, MAYV and CHIKV targeting different cells within the mosquito midgut [29], or to only a small number of cells that initially becomes infected in the midgut, making super infection exclusion during coinfections mostly irrelevant [29]. Depending on the time between infections with different viruses (sequential infections), it may be that the effect of SIE would be more profound, if e.g., the first infecting virus has already infected most midgut cells before entry of the second virus. Some sequential coinfection studies have found effects that support this. For example, CHIKV was excluded in MAYV-infected *Ae. aegypti* mosquitoes, but not the other way around [33], and *Culex pipiens* previously infected with USUV reduced transmission rate of West Nile virus [28].

Our study shows for the first time that UNAV can infect and replicate in *Ae. aegypti* mosquitoes. The virus was previously isolated from *Aedes serratus* and *Aedes leucocelaenus*, but has not yet been reported in *Ae. aegypti* to our knowledge [15]*.* Surprisingly, UNAV infection and transmission rates were more comparable with those of CHIKV than those of MAYV, even though UNAV is genetically more related to MAYV [39]. This study's CHIKV infection and transmission rates (94 % infection- and 12 % transmission rate) were higher than reported in a previous study using the same CHIKV strain, a similar viral infection titer and the same *Ae. aegypti* population (67 % infection rate and a 6 % transmission rate) [30]. UNAV had infection and transmission rates comparable to CHIKV in our study (76 % and 14 % respectively).

One study limitation may be that UNAV titers in the blood meals used to infect the mosquitoes were consistently higher than the MAYV and CHIKV blood titres in all treatments. This suggests that the starting conditions of the coinfection experiments were slightly skewed towards UNAV. This could potentially have resulted in UNAV dominating the coinfected mosquito samples, having a competitive advantage. We did not find evidence, however, for interference in our samples (Fig. 3C-E), so the titer difference seemed to have had little effect.

Another limitation was the fact that we were not able to detect viral RNA in all CPE-positive saliva samples with our qRT-PCR approach. This was probably the result of the different sensitivities of the two assays. Future studies could use a probe-based qRT-PCR design (e.g. TaqMan) as this is likely to be more robust and sensitive [28,29]. Using immunofluorescence to visualise viral proteins and study viral tropism in different tissues after coinfection could potentially answer the question why these viruses that use the same replication sites are not in apparent competition with each other.

Next to exploring the effects of coinfections on transmission under controlled conditions, we need to consider natural variation in our study system. First, humans may have different levels of viremia when exposed to an arbovirus. For example, ZIKV viremia is generally lower than CHIKV viremia [40]. Second, *Ae. aegypti* mosquitoes are known to take partial bloodmeals and have multiple feeding episodes, and third, virus dynamics are temperature dependent. These three factors will affect the amount of virus being ingested and the possible timing between two (or more) sequential infections. Moreover, viral kinetics may differ in geographical areas with varying temperatures [41]. It is known that successive blood meals can enhance virus dissemination within mosquitoes and increase transmission potential due to changes to the midgut epithelium [42], and a recent study found that temperature indeed affects the vectorial capacity of coinfected mosquitoes with MAYV and DENV [43]. Next to strict arboviruses, mosquitoes are also infected with a wide range of insect-specific viruses, for which we hardly know how they affect arbovirus infections [44]. Therefore, investigating the effects of these factors on the transmission potential of *Ae. aegypti* remains critical for understanding the bigger picture of the impact of coinfections.

As we show that *Ae. aegypti* is experimentally capable of transmitting UNAV, it may be beneficial for public health efforts to better characterise UNAV infections in humans and wild mosquito populations. This is important as susceptibility for arboviral infections varies across different geographic populations of *Ae. aegypti*, and varies within the same population with different viral species or strains [45]. Just like UNAV at this moment, ZIKV was considered to be an obscure virus at the turn of the century, and it caused only a handful of seemingly benign infections in Africa [46]. However, within just a couple of years, ZIKV caused multiple epidemics in South and Central America in 2014–2016. Due to the high number of infected people, the causal link between congenital disabilities and ZIKV infections, later known as congenital Zika syndrome, was uncovered, which showcases the importance, but also the challenge of early characterisation of disease caused by arboviruses. The reason UNAV has not been associated with notable outbreaks is likely multifactorial and could be due to the virus itself, pre-existing alphavirus immunity in the population, variation in the native mosquito species and genetic population differences, or insufficient epidemiological data [46].

## 5 Conclusion

In conclusion, understanding how coinfections affect the biology and transmission of arboviruses is essential for assessing public health risks. Beyond the risk of spillover of arboviruses from wildlife to humans, there is the often-overlooked risk of spillback (or reverse zoonosis), where viruses move from humans to wildlife, potentially establishing permanent sylvatic reservoirs—as seen with yellow fever virus (YFV), which, after being introduced to the Americas alongside *Ae. aegypti*, transitioned from urban outbreaks to a sylvatic cycle involving non-human primates [47]. This further illustrates that emerging *Ae. aegypti*-vectored arboviruses are not just a public health concern but a broader One Health challenge. Particularly as ecological and environmental changes will increasingly drive the geographical distributions of arboviruses, their potential vectors, and hosts in the future.

## Funding

Laboratory of Entomology, Wageningen University.

This publication was made possible by CTSA Grant Number UL1 TR001863 from the National Center for Advancing Translational Science (NCATS), a component of the National Institutes of Health (CBFV). The content is solely the responsibility of the authors and does not necessarily represent the official views of the NIH.

## CRediT authorship contribution statement

**Tessa M. Visser:** Writing – original draft, Visualization, Methodology, Investigation, Formal analysis, Conceptualization. **Haidong D. Wang:** Writing – review & editing, Methodology, Formal analysis. **Sandra R. Abbo:** Writing – review & editing, Investigation. **Chantal B.F. Vogels:** Writing – review & editing, Conceptualization. **Constantianus J.M. Koenraadt:** Writing – review & editing, Funding acquisition, Conceptualization. **Gorben P. Pijlman:** Writing – review & editing, Funding acquisition, Conceptualization.

## Declaration of competing interest

Chantal Vogels reports financial support was provided by National Institutes of Health. If there are other authors, they declare that they have no known competing financial interests or personal relationships that could have appeared to influence the work reported in this paper.

## Data Availability

No data was used for the research described in the article.

## Appendix A Supplementary data

Supplementary material Image 1
