# Supplementary material for: Effect of chikungunya, Mayaro and Una virus coinfection on vector competence of Aedes aegypti mosquitoes
Source: One Health. 2025 Feb 7;20:100991. doi: 10.1016/j.onehlt.2025.100991 (PMC11869603; doi:10.1016/j.onehlt.2025.100991)
Supplement: Supplementary file 1 — Supplementary material [file mmc1.docx]

**Supporting information**

**Effect of chikungunya, Mayaro and Una virus coinfection on vector competence of *Aedes aegypti* mosquitoes**

**Authors:** Tessa M Visser^1*^, Haidong D Wang^2^, Sandra R Abbo^2^, Chantal BF Vogels^3^, Constantianus JM Koenraadt^1^, Gorben P Pijlman^2*^

**Affiliations:**^1^Laboratory of Entomology, Wageningen University and Research, Wageningen, The Netherlands
^2^Laboratory of Virology, Wageningen University and Research, Wageningen, The Netherlands
^3^Department of Epidemiology of Microbial Diseases, Yale School of Public Health, New Haven, USA

**Present address:**^a^School of Public Health and Emergency Management, South University of Science and Technology (SUSTech), Shenzhen, China
^b^Section Virology, Division Infectious Diseases and Immunology, Department of Biomolecular Health Sciences, Faculty of Veterinary Medicine, Utrecht University, Utrecht, the Netherlands

*Corresponding authors, email: [tessa.visser@wur.nl](mailto:tessa.visser@wur.nl); gorben.pijlman@wur.nl

**Table S1: Primers used for qPCR.** Primers were designed based on the E1 envelope gene for all three viruses. For UNAV and MAYV degenerate primers were designed first on sequences from GenBank, and used to sequence part of the E1 gene of each virus, next specific primers were designed based on the received sequences. CHIKV primers were designed solely using the GenBank sequence.

| Primers | Genome position | Sequence 5’ to 3’ | Base pair length | GenBank accession degenerate primer design |  |
| --- | --- | --- | --- | --- | --- |
| CHIKV qF | E1, 11.061 | TTCTCAACAGCCCTGGCAAG | 190 | EU224270.1 | |
| CHIKV qR | E1, 11.231 | ATCCTACTCCTCCCGTAATC |  |  | |
| T7 CHIKV F | E1 | **TAATACGACTCACTATAGGG**TTCTCAACAGCCCTGGCAAG | 210 |  | |
| MAYV qF | E1, 10.721 | TCTCTGAGTTGACGTGCACC | 210 | MK573238.1 | |
| MAYV qR | E1, 10.911 | AAACTACGAAGGAAGGGGCG |  |  | |
| T7 MAYV F | E1 | **TAATACGACTCACTATAGGG**TCTCTGAGTTGACGTGCACC | 230 |  | |
| UNAV qF | E1, 10.659 | CCGGTAGGTTCGGTGACATC | 125 | HM147992.1 | |
| UNAV qR | E1, 10.783 | GGAAGGGGTCTGTGTGTACG |  |  | |
| T7 UNAV F | E1 | **TAATACGACTCACTATAGGG**CCGGTAGGTTCGGTGACATC | 145 |  | |

**Table S2: Specificity of the SYBR qPCR system.** In almost all cases the other viruses were also picked-up in our assay at a very high Ct indicating some cross-reactivity between the primers and different RNA sources. Therefore, melt-curve temperatures were determined for each sample.

| **Dilution of UNAV RNA** |  | *Ct* |  |  |
| --- | --- | --- | --- | --- |
| **ng/reaction** | **genome copies/reaction** | **UNAV** | **MAYV** | **CHIKV** |
| 7.82E+00 | 1.01E+11 | 12.63 | 33.80 | 29.01 |
| 7.82E-01 | 1.01E+10 | 17.155 | 37.56 | 32.00 |
| 7.82E-02 | 1.01E+09 | 20.365 | 37.01 | 36.46 |
| 7.82E-03 | 1.01E+08 | 23.685 | 38.32 | 37.34 |
| 7.82E-04 | 1.01E+07 | 25.84 | N/A | 35.35 |
| 7.82E-05 | 1.01E+06 | 29.175 | 39.87 | 38.32 |
| 7.82E-06 | 1.01E+05 | 32.855 | 39.33 | 37.19 |
| 7.82E-07 | 10093.75 | 36.51 | 37.83 | 37.02 |
| H2O | x | N/A | 36.42 | 38.06 |
|  |  |  |  |  |
| **Dilution of MAYV RNA** |  | *Ct* |  |  |
| **ng/reaction** | **genome copies/reaction** | **MAYV** | **UNAV** | **CHIKV** |
| 1.25E+00 | 1.02E+10 | 14.405 | 33.51 | 31.815 |
| 1.25E-01 | 1.02E+09 | 17.87 | 30.39 | 36.24 |
| 1.25E-02 | 1.02E+08 | 20.225 | 36.56 | 35.32 |
| 1.25E-03 | 1.02E+07 | 24.295 | N/A | 36.38 |
| 1.25E-04 | 1.02E+06 | 27.435 | N/A | 36.41 |
| 1.25E-05 | 1.02E+05 | 31.8 | N/A | N/A |
| 1.25E-06 | 10202.94 | 35.54 | N/A | N/A |
| 1.25E-07 | 1020.29 | 38.555 | 37.33 | 35.46 |
| H2O | x | 39.97 | 38.23 | 35.31 |
|  |  |  |  |  |
| **Dilution of CHIKV RNA** |  | *Ct* |  |  |
| **ng/reaction** | **genome copies/reaction** | **CHIKV** | **UNAV** | **MAYV** |
| 1.11E+00 | 9.94E+09 | 10.165 | N/A | 37.26 |
| 1.11E-01 | 9.94E+08 | 14.125 | N/A | 39.22 |
| 1.11E-02 | 9.94E+07 | 23.86 | N/A | 32.75 |
| 1.11E-03 | 9.94E+06 | 22.215 | N/A | 36.54 |
| 1.11E-04 | 9.94E+05 | 24.76 | N/A | 37.855 |
| 1.11E-05 | 9.94E+04 | 28.995 | 33.63 | 37.965 |
| 1.11E-06 | 9937.50 | 32.995 | N/A | 37.97 |
| 1.11E-07 | 993.75 | 37.375 | 36.77 | N/A |
| H2O | x | N/A | N/A | 38.85 |

**Table S3: Summary of infection and transmission rates of all experiments based on CPE.**

| **Treatment** | **Positive bodies** | **Positive saliva's** | **Total mosquito #** | | **Infection %** | **SEM** | **Transmission %** | **SEM** |
| --- | --- | --- | --- | --- | --- | --- | --- | --- |
| UNAV | 73 | 13 | 96 | 76,04 | | 6,02 | 13,54 | 0,97 |
| MAYV | 51 | 12 | 89 | 57,30 | | 1,73 | 13,48 | 4,63 |
| CHIKV | 97 | 12 | 103 | 94,17 | | 1,62 | 11,65 | 1,52 |
| UNAV/MAYV | 62 | 14 | 78 | 79,49 | | 3,92 | 17,95 | 2,92 |
| UNAV/CHIKV | 59 | 9 | 70 | 84,29 | | 3,61 | 12,86 | 6,84 |
| MAYV/CHIKV | 46 | 9 | 68 | 67,65 | | 12,26 | 13,24 | 5,54 |
| UNAV/MAYV  /CHIKV | 48 | 6 | 63 | 76,19 | | 9,45 | 9,52 | 3,18 |

**Table S4: The single experiment infection and transmission results per replicate based on CPE.**

| **Treatment** | **Replicate** | **Positive bodies** | **Positive saliva's** | **Total mosquito #** | **Infection %** | **SEM** | **Transmission %** | **SEM** |
| --- | --- | --- | --- | --- | --- | --- | --- | --- |
| UNAV | 1 | 22 | 3 | 25 | 88,00 | 6,02 | 12,00 | 0,97 |
| UNAV | 2 | 9 | 2 | 15 | 60,00 |  | 13,33 |  |
| UNAV | 3 | 22 | 5 | 31 | 70,97 |  | 16,13 |  |
| UNAV | 4 | 20 | 3 | 25 | 80,00 |  | 12,00 |  |
| MAYV | 1 | 5 | 1 | 8 | 62,50 | 1,73 | 12,50 | 4,63 |
| MAYV | 2 | 12 | 5 | 20 | 60,00 |  | 25,00 |  |
| MAYV | 3 | 16 | 5 | 28 | 57,14 |  | 17,86 |  |
| MAYV | 4 | 18 | 1 | 33 | 54,55 |  | 3,03 |  |
| CHIKV | 1 | 14 | 2 | 18 | 77,78 | 1,62 | 11,11 | 1,52 |
| CHIKV | 2 | 14 | 3 | 19 | 73,68 |  | 15,79 |  |
| CHIKV | 3 | 25 | 4 | 31 | 80,65 |  | 12,90 |  |
| CHIKV | 4 | 26 | 3 | 35 | 74,29 |  | 8,57 |  |

**Table S5: The duo and triple experiment infection and transmission results per replicate based on CPE.**

| **Treatment** | **Replicate** | **Positive bodies** | **Positive saliva's** | **Total mosquito #** | **Infection %** | **SEM** | **Transmission%** | **SEM** |
| --- | --- | --- | --- | --- | --- | --- | --- | --- |
| UNAV/MAYV | 1 | 37 | 10 | 44 | 84,09 | 3,92 | 22,73 | 2,92 |
| UNAV/MAYV | 2 | 4 | 1 | 6 | 66,67 |  | 16,67 |  |
| UNAV/MAYV | 3 | 14 | 2 | 18 | 77,78 |  | 11,11 |  |
| UNAV/MAYV | 4 | 7 | 1 | 10 | 70,00 |  | 10,00 |  |
| UNAV/CHIKV | 1 | 31 | 3 | 35 | 88,57 | 3,61 | 8,57 | 6,84 |
| UNAV/CHIKV | 2 | 6 | 3 | 8 | 75,00 |  | 37,50 |  |
| UNAV/CHIKV | 3 | 14 | 2 | 18 | 77,78 |  | 11,11 |  |
| UNAV/CHIKV | 4 | 8 | 1 | 9 | 88,89 |  | 11,11 |  |
| MAYV/CHIKV | 1 | 32 | 8 | 43 | 74,42 | 12,26 | 18,60 | 5,54 |
| MAYV/CHIKV | 2 | 2 | 0 | 6 | 33,33 |  | 0,00 |  |
| MAYV/CHIKV | 3 | 12 | 1 | 19 | 63,16 |  | 5,26 |  |
| UNAV/MAYV/CHIKV | 1 | 30 | 3 | 34 | 88,24 | 9,45 | 8,82 | 3,18 |
| UNAV/MAYV/CHIKV | 2 | 3 | 0 | 7 | 42,86 |  | 0,00 |  |
| UNAV/MAYV/CHIKV | 3 | 5 | 1 | 8 | 62,50 |  | 12,50 |  |
| UNAV/MAYV/CHIKV | 4 | 10 | 2 | 14 | 71,43 |  | 14,29 |  |

**Table S6: PubMed literature review of the distribution of UNA, MAYV and CHIKV on the South American continent.** Literature sources include reported human cases, and detection in mosquitoes and non-human primates.

| **Countries** | **Una virus** | **Mayaro virus** | **Chikungunya virus** |
| --- | --- | --- | --- |
| Argentina | [1–3] |  | [4] |
| Bolivia |  | [3,5] | [4] |
| Brazil | [3] | [3,5] | [4] |
| Chile |  |  | [4] |
| Colombia | [3] | [5] | [4] |
| Ecuador |  | [5] | [4] |
| French Guiana (France) |  | [3,5] | [4] |
| Guyana |  |  | [4] |
| Panama | [6] | [5,6] | [4] |
| Paraguay | [1,7] |  | [4] |
| Peru | [3,8] | [3,5,8] | [4] |
| Trinidad & Tobago | [3] | [3,5] | [4] |
| Suriname |  | [3,5] | [4] |
| Uruguay |  |  | [4] |
| Venezuela | [3] | [5] | [4] |

Literature list

1. Díaz LA, del Pilar Díaz M, Almirón WR, Contigiani MS. Infection by UNA virus (Alphavirus; Togaviridae) and risk factor analysis in black howler monkeys (*Alouatta caraya*) from Paraguay and Argentina. Trans R Soc Trop Med Hyg. 2007;101: 1039–1041. doi:10.1016/j.trstmh.2007.04.009

2. Díaz LA, Spinsanti LI, Almirón WR, Contigiani MS. UNA virus: first report of human infection in Argentina. Rev Inst Med Trop Sao Paulo. 2003;45: 109–110. doi:10.1590/S0036-46652003000200012

3. Powers AM, Aguilar P v., Chandler LJ, Brault AC, Meakins TA, Watts D, et al. Genetic relationships among Mayaro and Una viruses suggest distinct patterns of transmission. Am J Trop Med Hyg. 2006;75: 461–469. doi:10.4269/AJTMH.2006.75.461

4. PAHO / WHO Health Information Platform for the Americas (PLISA). Cases of Chikungunya Virus Disease. [cited 14 Dec 2022]. Available: https://bit.ly/3We3TSu

5. Diagne CT, Bengue M, Choumet V, Hamel R, Pompon J, Missé D. Mayaro virus pathogenesis and transmission mechanisms. Pathogens. MDPI AG; 2020. pp. 1–23. doi:10.3390/pathogens9090738

6. Carrera JP, Cucunuba ZM, Neira K, Lambert B, Pitti Y, Liscano J, et al. Endemic and epidemic human Alphavirus infections in eastern Panama: an analysis of population-based cross-sectional surveys. Am J Trop Med Hyg. 2020;103: 2429–2437. doi:10.4269/AJTMH.20-0408

7. Cardozo F, Konigheim B, Albrieu-Llinás G, Rivarola ME, Aguilar J, Rojas A, et al. Alphaviruses: serological evidence of human infection in Paraguay (2012-2013). Vector-Borne and Zoonotic Diseases. 2018;18: 266–272. doi:10.1089/vbz.2017.2178

8. Pérez JG, Carrera JP, Serrano E, Pittí Y, Maguiña JL, Mentaberre G, et al. Serologic evidence of zoonotic Alphaviruses in humans from an indigenous community in the Peruvian Amazon. Am J Trop Med Hyg. 2019;101: 1212–1218. doi:10.4269/AJTMH.18-0850

**Figure S1: Amplification efficiency and standard curves of UNAV, MAYV and CHIKV primers.** The efficiencies were calculated on single RNA sample and triple RNA sample curves containing all three viruses, for (**A**) UNAV in orange, (**B**) MAYV in green and (**C**) CHIKV in blue. The single RNA standard curves can be seen together in (**D**). The in vitro transcript viral RNA was quantified by the DeNovix spectrophotometer, and used to make a 10-time dilution series. The viral genome copies were calculated by an online tool: <https://nebiocalculator.neb.com/>.
